# How adenomyosis changes throughout pregnancy: A retrospective cohort study

**DOI:** 10.1002/ijgo.14383

**Published:** 2022-08-17

**Authors:** Emma Bertucci, Filomena G. Sileo, Marialaura Diamanti, Carlo Alboni, Fabio Facchinetti, Antonio La Marca

**Affiliations:** ^1^ Prenatal Medicine Unit, Obstetrics and Gynecology Unit, Department of Medical and Surgical Sciences for Mother, Child and Adult University of Modena and Reggio Emilia Modena Italy; ^2^ Department of Biomedical, Metabolic and Neural Sciences International Doctorate School in Clinical and Experimental Medicine, University of Modena and Reggio Emilia Modena Italy; ^3^ Unit of Obstetrics and Gynecology Azienda Unità Sanitaria Locale—IRCCS Reggio Emilia Italy; ^4^ Obstetrics and Gynecology Unit, Department of Medical and Surgical Sciences for Mother, Child and Adult University of Modena and Reggio Emilia Modena Italy

**Keywords:** adenomyosis, pregnancy, sonography, uterus

## Abstract

**Objective:**

To study how adenomyosis changes during pregnancy and to possibly correlate these changes to maternal and fetal outcomes.

**Methods:**

Retrospective exploratory cohort study including 254 women with a pre‐conceptional/first‐trimester scan to document adenomyosis and known obstetric outcome. If visible, adenomyosis signs were documented in each trimester and postpartum. Mann–Whitney *U* tests or χ^2^ tests were used for continuous and categorical variables, respectively.

**Results:**

A globular uterus was reported in 79% (*n* = 52) of women with adenomyosis in the first trimester, in 38% (*n* = 20) and 2% (*n* = 1) of women in the second and third trimesters, respectively, and postpartum in 77% (*n* = 34) of women. Asymmetrical thickening (*n* = 20, 30%) and cysts (*n* = 15, 23%) were only visible in 1st trimester. Adenomyosis was associated with miscarriage (odds ratio [OR] 5.9, 95% confidence interval [CI] 2.4–14.9, *P* < 0.001) also in normal conception only (OR 5.1, 95% CI 1.8–14.2, *P* = 0.002) or adjusting for maternal age (adjusted OR 5.9, 95% CI 2.3–15.2, *P* < 0.001). Gestational age at delivery was lower in adenomyosis (*P* = 0.004); the cesarean section rate was higher than in controls (OR 2.5, 95% CI 1.3–4.8, *P* = 0.007) also adjusting for age (adjusted OR 2.07, 95% CI 1.06–4.08, *P* = 0.035).

**Conclusions:**

Signs of adenomyosis were visible but progressively disappeared in pregnancy; adenomyosis was associated with an increased risk of early miscarriage. Prospective studies are needed to confirm our results.

## Synopsis

Sonographic signs of adenomyosis are visible in pregnancy although they progressively disappear; adenomyosis was associated with an increased risk of early miscarriage*.*

## 1 INTRODUCTION

Adenomyosis is characterized by the localization of the endometrial glands and stroma in the myometrial wall of the uterus with reactive hyperplasia and fibrosis of the surrounding myometrial smooth muscle cells^1^, ^2^ with a reported prevalence between 5%–35% and 14%–66%.^3^, ^4^, ^5^

Women with uterine adenomyosis may be asymptomatic (35%) or report menorrhagia (50%), dysmenorrhea (30%), and metrorrhagia (20%).^6^ Although usually considered a pathology of parous women, several authors recently linked this condition to subfertility because many women with "unexplained infertility" were found to have adenomyosis.^7^

Despite a reported prevalence of adenomyosis of 38.2% in cases of recurrent pregnancy loss and of 34.7% in previous assisted reproductive technology failure,^8^ less attention was given to the potential effects of adenomyosis on pregnancy outcomes. Adenomyosis was associated with an increased risk of preterm birth and preterm prelabor rupture of membranes,^9^, ^10^ second‐trimester miscarriage, pre‐eclampsia, placental malposition,^10^ and small‐for‐gestational‐age (SGA) infants.^11^

Being relatively inexpensive and accurate, ultrasound is now considered the imaging modality of choice for diagnosing adenomyosis. In fact, using the consensus‐based practical sonographic classification of adenomyosis^12^ and the Morphological Uterus Sonographic Assessment criteria,^13^ the identification of adenomyosis relies on seven sonographic aspects: identification and determination of location of adenomyosis; differentiation between focal and diffuse disease and between cystic and non‐cystic lesion; determination of myometrial layer involvement; classification of disease extent as mild, moderate, or severe; and measurement of size of lesion.^12^

To our knowledge there are no data on the appearance and the possible modifications of adenomyosis during pregnancy: the main objective of this study was to evaluate how ultrasound characteristics of adenomyosis modify during pregnancy and to possibly correlate these changes to maternal and fetal outcomes.

## 2 MATERIALS AND METHODS

This is a retrospective exploratory study conducted between 2016 and 2020 in a University Hospital.

Inclusion criteria were: women aged more than 18 years of age, singleton pregnancy, available recorded ultrasound imaging in pregnancy and known outcomes. In particular, women were included in the study if at least one transvaginal scan (before conception or during the first trimester) was available to assess the presence of adenomyosis and if at least one scan per trimester (if ongoing pregnancy) and the complete information of maternal and neonatal outcomes were available. Being a referral center for endometriosis and adenomyosis, sonographic signs of adenomyosis are usually assessed and recorded during gynecologic or early (<12 weeks of gestation) pregnancy scans.

We excluded multiple pregnancies, women diagnosed with uterine malformations or fetal anomalies.

Due to the retrospective nature of the study, no informed consent was required. The study has been approved by the local Ethics Committee (approval number 1315/2020/OSS*/AOUMO). Patients were not involved in the development of this research; core outcome sets were not used; and the local Ethics Committee approved the use of anonymized data in this study.

### 2.1 Study protocol

Women with at least one transvaginal scan recorded before conception or during the first trimester were assessed for eligibility and included if at least one scan per trimester (for ongoing pregnancies) and all information about obstetric and neonatal outcomes could be retrieved from clinical records.

Women were diagnosed as having adenomyosis at the first transvaginal scan according to the Morphological Uterus Sonographic Assessment classification and the recent consensus classification system for adenomyosis.^12^, ^13^

Adenomyosis was defined as focal in the presence of adenomyosis‐related lesions in only one part of the myometrium, or as diffuse in the presence of lesions in more than one site or dispersed within the myometrium.^13^

The following sonographic signs for adenomyosis were evaluated for each patient in each trimester and postpartum (1–3 months after delivery): globular aspect of the uterus; presence of adenomyoma; presence of hyperechoic islands; fan‐shaped shadowing; asymmetrical thickening of the myometrial walls, with either increased or decreased echogenicity; presence of cystic structure in the myometrium; echogenic subendometrial lines and buds; translesional vascularity. A precise localization of all sonographic signs, when present, was reported in all women and in subsequent scans, the same operator (EB) assessed if these signs were still detectable at ultrasound.

The involvement of the uterine junctional zone was not evaluated because a three‐dimensional scan before the pregnancy was not available for all patients and its evaluation across the pregnancy was not reliable because of pregnancy‐induced modification of the junctional zone itself.^14^

Two authors (EB and FGS) reviewed retrospectively all available images for each patient.

### 2.2 Variables of interest

General characteristics, obstetric and neonatal outcomes were retrieved from medical records. Among the obstetric and neonatal outcomes we evaluated: incidence of miscarriage, SGA defined as birth weight below the 10th centile according to Neonatal Italian Charts (INES Charts),^15^ intrauterine fetal demise (IUFD) defined as death after 24 weeks of pregnancy, admission to Neonatal Intensive Care Unit (NICU) and adverse obstetric outcome (defined as at least one among: miscarriage, IUFD, SGA, and/or NICU admission). Karyotype investigation of miscarriages was performed and recorded if clinically indicated. We also evaluated: the incidence of preterm birth, i.e. spontaneous or iatrogenic birth occurring before 37 weeks, mode of birth (i.e. vaginal delivery, vacuum extraction, elective or urgent cesarean section [CS]), operative delivery (defined as vacuum extraction or urgent CS), blood loss at birth, and incidence of postpartum hemorrhage, i.e. blood loss of at least 500 ml or at least 1000 ml at vaginal birth or CS, respectively, and incidence of retained placental remnants requiring surgical removal.

### 2.3 Statistical analysis

Continuous variables were presented as median (interquartile range); binary and categorical variables were presented as numbers and percentages. Continuous variables were compared using *t* test or Mann‐Whitney *U* tests according to normality, while the χ^2^ or Fisher exact tests were used for binary or categorical variables. A value of *P* less than 0.05 was considered statistically significant. Analyses were performed using SPSS Version 21 (IBM).

## 3 RESULTS

During the study period, 254 women were included; 66 patients were diagnosed as having adenomyosis: half of them presented with focal (*n* = 33) adenomyosis while the remaining 33 patients exhibited diffuse adenomyosis. There were no differences between women with a diagnosis before or within the first 12 weeks of pregnancy (data not shown). The remaining 188 women were used as controls.

**FIGURE 1:**
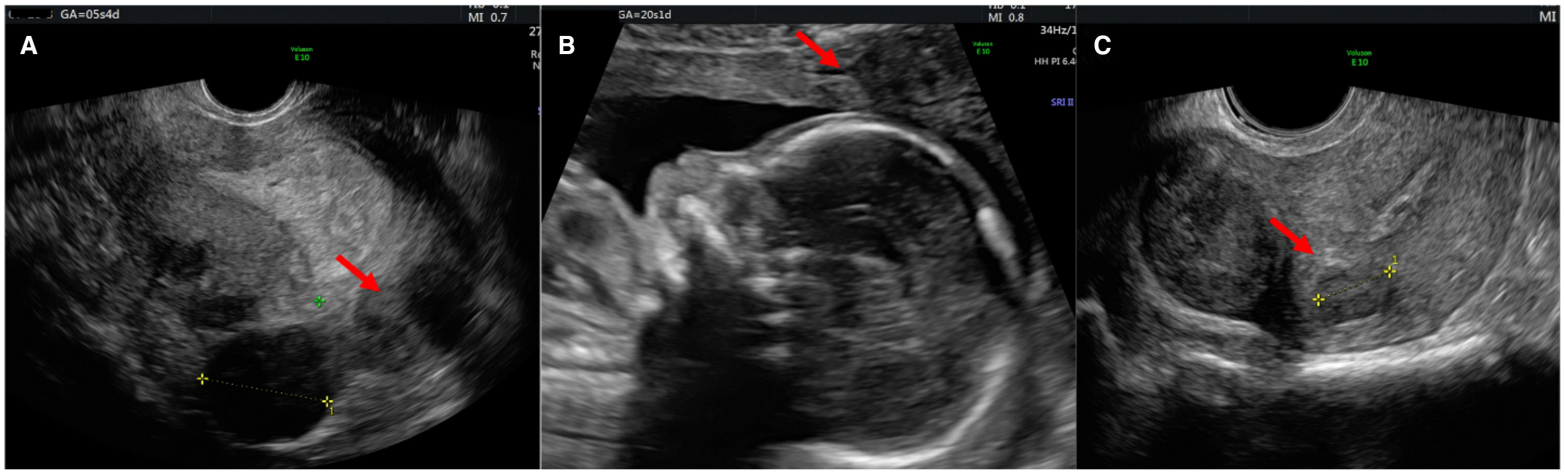
Presence of adenomyoma (red arrows) at 5 weeks of pregnancy (a), at 20 weeks (b), and postpartum (c)

**FIGURE 2:**
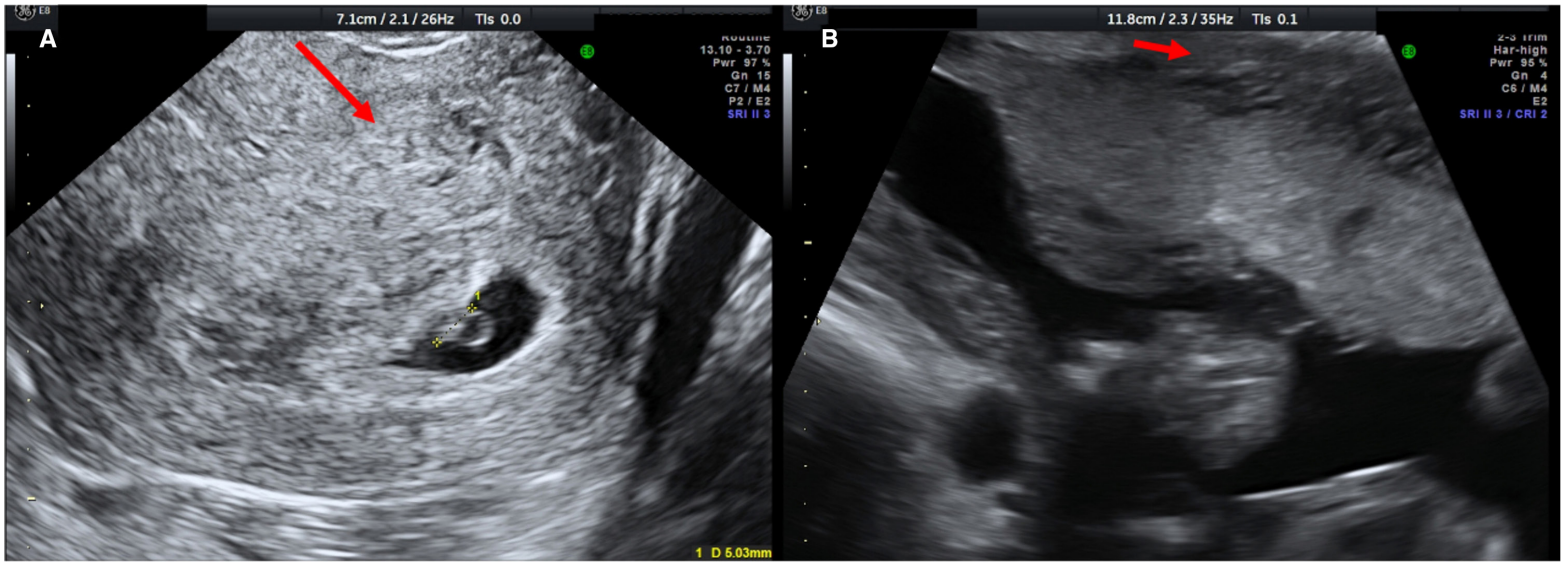
Presence of hyperechoic islands (red arrows) at 5 weeks (a) and at 18 weeks (b)

**FIGURE 3:**
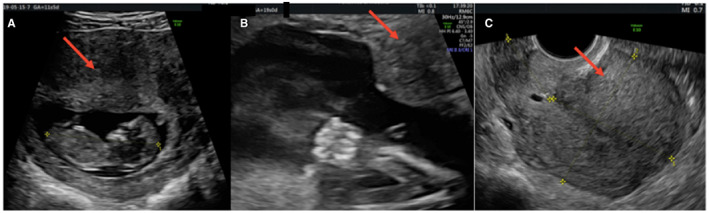
Presence of globular aspect of the uterus (red arrows) at 12 weeks (a), at 19 weeks (b), and postpartum (c)

**FIGURE 4:**
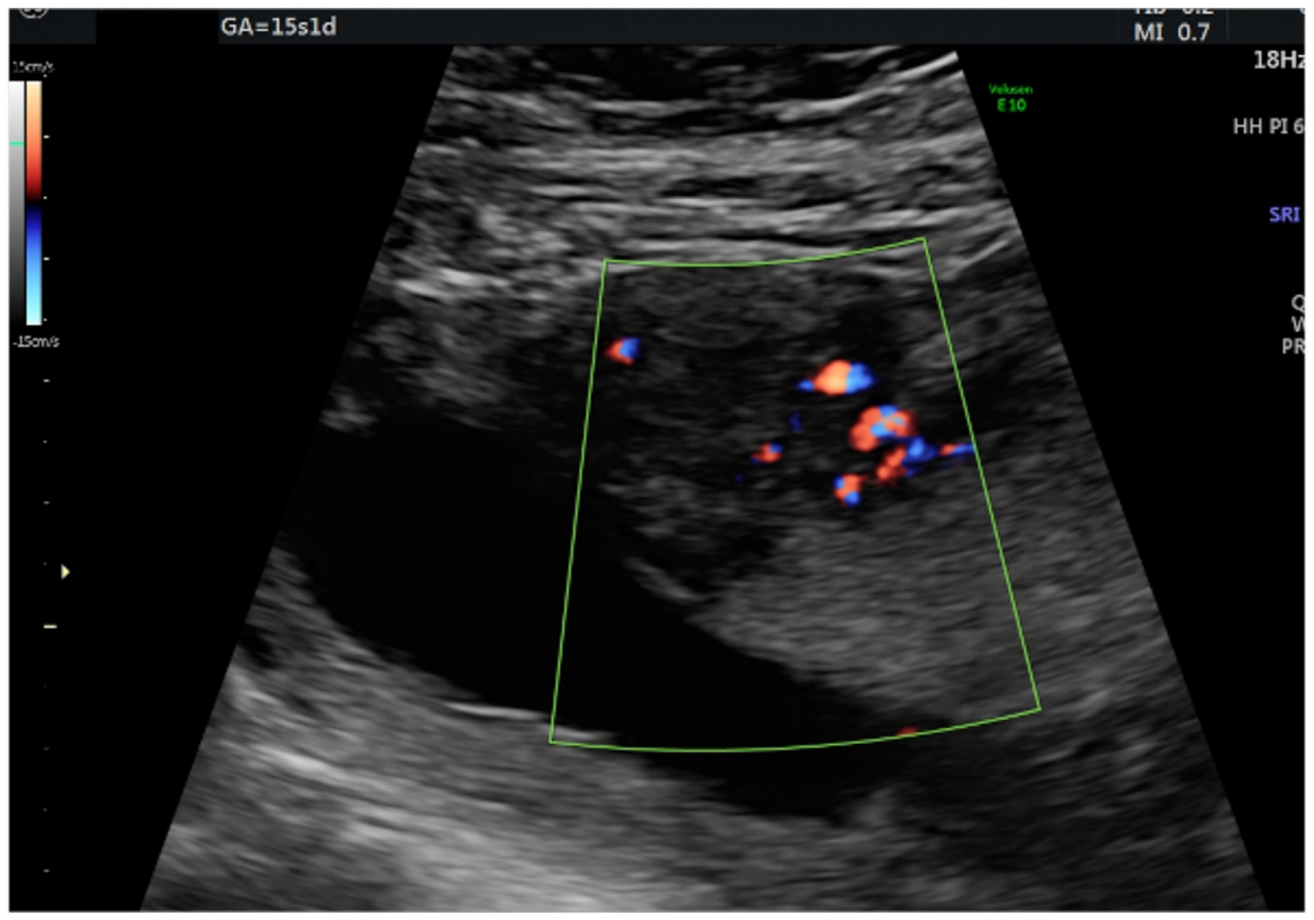
Appearance of the translesional vascularity at 15 weeks

**FIGURE 5:**
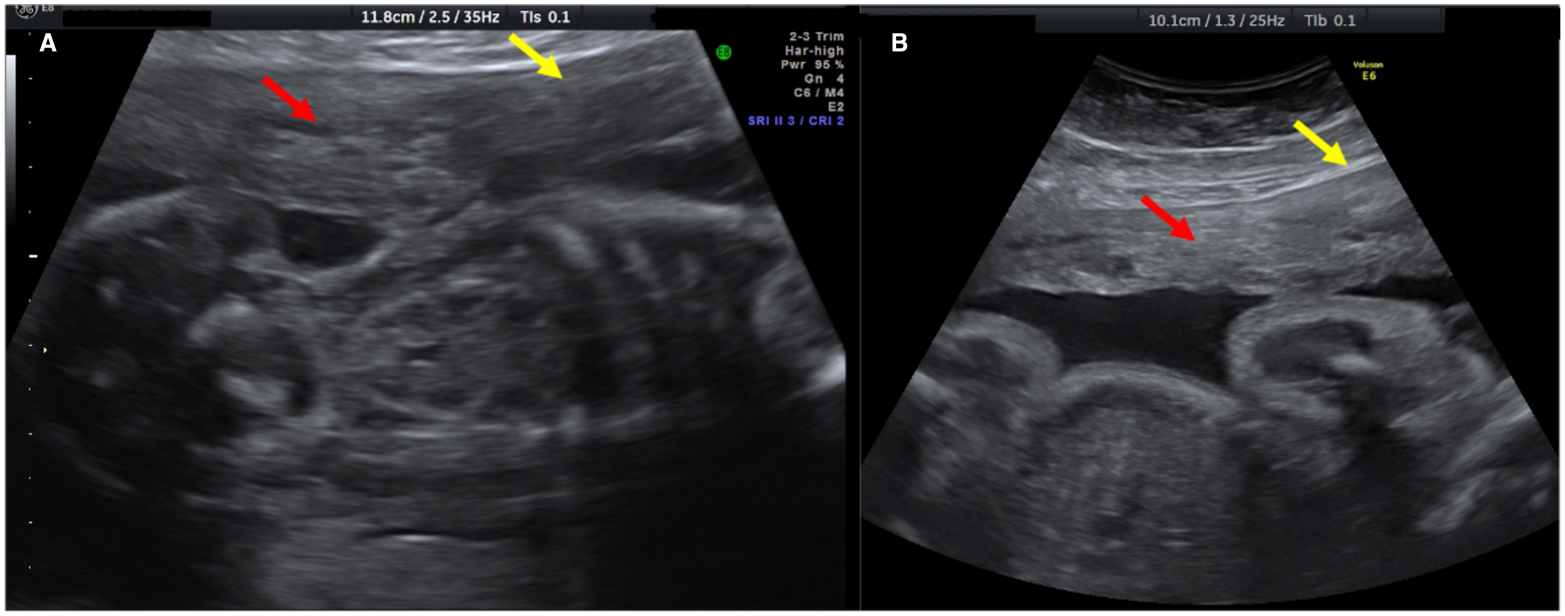
Presence of both hyperechoic islands (red arrows) and asymmetrical thickness (yellow arrows) in the same patient at 20 weeks (a) and at 30 weeks (b)

In Figures 1, 2, 3, 4, 5, signs of adenomyosis at different gestational ages or postpartum are presented.

The distributions of the different signs according to trimesters and in the postpartum period (1–3 months after delivery) are presented in Table 1. In particular, 66 women diagnosed with adenomyosis were available for the first‐trimester evaluation. After excluding 14 women experiencing a spontaneous miscarriage in the first trimester, 52 women were available for evaluation in the second and third trimesters. Finally, 44 women were available for postpartum evaluation because eight women were lost to follow up.

**TABLE 1:** Distribution of signs according to the trimester of pregnancy and in the postpartum period^a^

	First trimester (*n* = 66)	Second trimester (*n* = 52)^b^	Third trimester (*n* = 52)^b^	Postpartum (*n* = 44)^c^
Globular aspect	52 (79)	20 (38)	1 (2)	34 (77)
Adenomyoma	2 (3)	1 (2)	0 (0)	0 (0)
Hyperechoic islands	3 (5)	1 (2)	0 (0)	4 (9)
Fan‐shaped shadowing	2 (3)	0 (0)	0 (0)	0 (0)
Asymmetrical thickening	20 (30)	4 (8)	0 (0)	14 (32)
Cysts	15 (23)	10 (19)	1 (2)	7 (16)
Echogenic subendometrial lines and buds	0 (0)	0 (0)	0 (0)	0 (0)
Translesional vascularity	1 (1.5)	1 (1.9)	0 (0)	0 (0)

^a^ Data are presented as number (percentage).

^b^ Women available for evaluation after excluding those having a spontaneous abortion in the first trimester (*n* = 14).

^c^ Women available for evaluation postpartum.

When looking at the signs, the globular aspect of the uterus was the most commonly (52; 79%) reported sign in the first trimester, which progressively disappeared as pregnancy progressed (20 [38%] and 1 [2%] of patients in second and third trimesters, respectively) and then became visible again in the postpartum period (34; 77%). The other most commonly reported signs in the first trimester were the asymmetrical thickening (20; 30%) and the cysts (15; 23%), which both became less visible in the second and third trimesters and became visible again postpartum (14 [32%] and 7 [16%], respectively). All the other signs were rarely seen in the first trimester and throughout the whole pregnancy. Echogenic subendometrial lines and buds were not recorded among women with adenomyosis.

Women with or without adenomyosis differed on mean maternal age (*P* = 0.011) and mean body mass index (BMI; calculated as weight in kilograms divided by the square of height in meters; *P* = 0.017) (see Table S1). Four (1.6%) women opted for termination of pregnancy and were excluded from further analysis.

Adenomyosis was significantly associated with adverse obstetric outcome (odds ratio [OR] 2.75, 95% confidence interval [CI] 1.44–5.20, *P* = 0.003): when considering adverse obstetric outcomes individually, it remained associated only with miscarriages in the first trimester (OR 5.92, 95% CI 2.36–14.89, *P* < 0.001), also when considering only women with normal conception (OR 5.10, 95% CI 1.83–14.22, *P* < 0.001) (see Table 2). This association between adenomyosis and miscarriage was confirmed when correcting for age (adjusted OR 5.99, 95% CI 2.34–15.15, *P* < 0.001) but not for BMI (*P* = 0.17). Karyotype was performed only in three cases of miscarriage.

**TABLE 2:** Obstetric and neonatal outcomes of the population according to the presence or absence of adenomyosis^a^

	Adenomyosis	No adenomyosis	*P* value
Obstetric adverse outcome^b^ (*n* = 250)	23 (34.8)	30 (16.3)	0.002
Spontaneous abortion (*n* = 50)	14 (21.2)	8 (4.3)	<0.001
Spontaneous abortion after normal conception (*n* = 209)	10 (19.2)	7 (4.5)	<0.001
GA at delivery, week	39.0 (38.3–39.6)	39. 6 (38.7–40.3)	0.004
Preterm delivery (<37 week), (*n* = 226)	6 (12)	8 (4.5)	0.054
Birth weight, g	3230 (2795–3545)	3260 (2930–3520)	0.675
SGA (*n* = 226)	5 (10.2)	19 (10.8)	0.906
NICU admission (*n* = 226)	4 (8.3)	4 (2.3)	0.069
CS (*n* = 226)	21 (42)	40 (22.7)	0.007
CS (no previous CS) (*n* = 200)	15 (34.9)	29 (18.5)	0.021
Operative delivery (*n* = 226)	8 (16)	23 (13.1)	0.596
Amount of bleeding, ml	350 (200–650)	300 (200–500)	0.249
PPH (*n* = 226)	11 (22)	37 (21)	0.881
Placental remnant removal (*n* = 224)	11 1 (2.12)	39 4 (22.22.3)	0.937

Abbreviations: CS, cesarean section; GA, gestational age; IUFD, intrauterine fetal death; NICU, neonatal intensive care unit; PPH, postpartum hemorrhage; SGA, small for gestational age.

^a^ Data are presented as number (percentage), or as median (interquartile range).

^b^ Defined as at least one among: miscarriage, IUFD, SGA and/or NICU admission.

**FIGURE 6:**
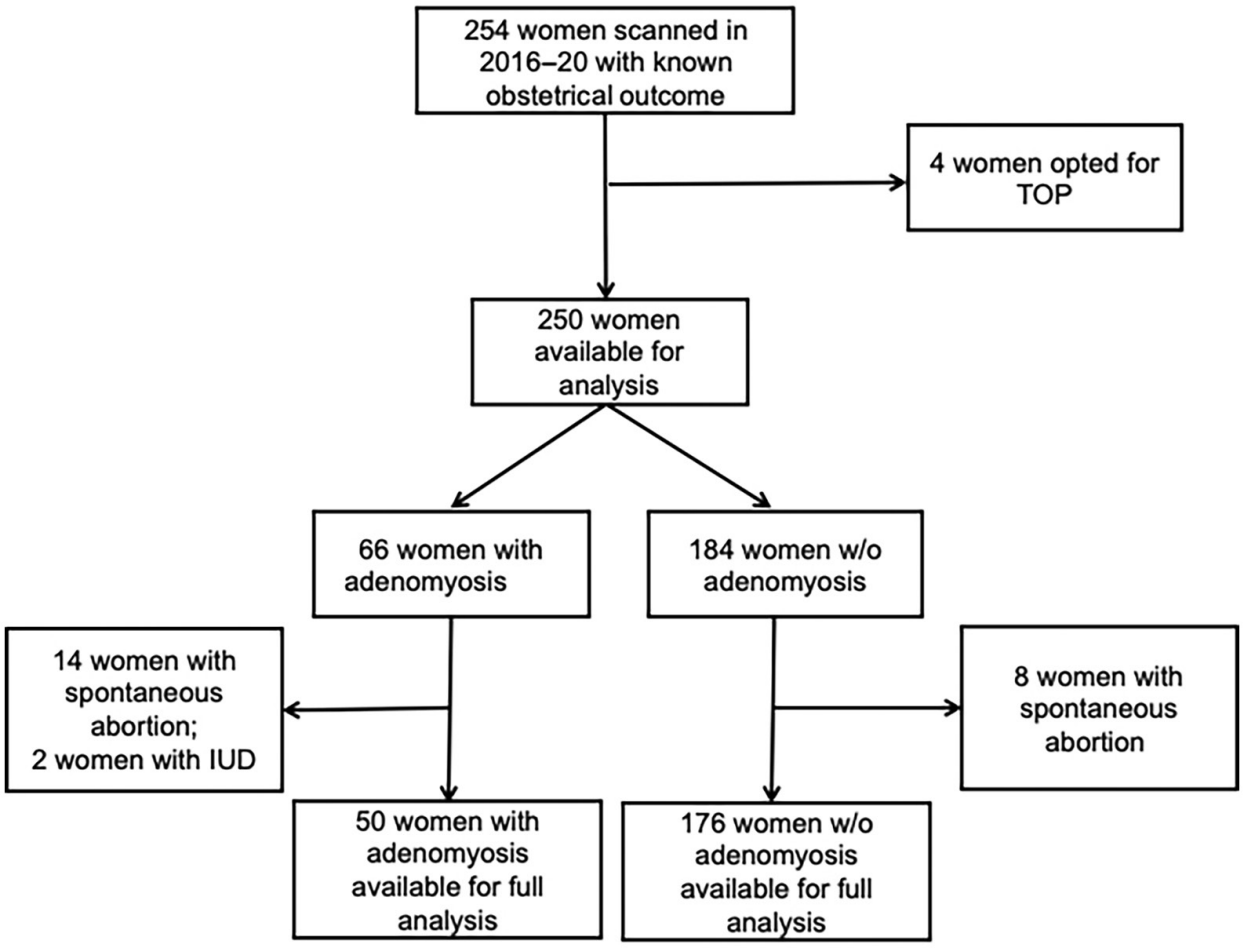
Flow chart of the patients and how they were included in the analysis

We did not test the possible association with IUFD and adenomyosis individually because of its low incidence (*n* = 2, 0.8%). The two IUFD and 22 miscarriages (8.8%) were then excluded from further analysis, leaving 226 women (see Figure 6).

Table 2 shows the distribution of obstetric and neonatal outcomes between the two groups.

Gestational age at delivery was significantly lower in women with adenomyosis compared with controls (*P* = 0.004), but the rate of preterm birth was not significantly higher in women with adenomyosis (*P* = 0.054).

In women with adenomyosis, the number of CS (urgent or elective) was higher than in controls (OR 2.46, 95% CI 1.27–4.78, *P* = 0.007) and this was confirmed (OR 2.37, 95% CI 1.12–4.98, *P* = 0.021) also after excluding all women with a previous CS (26/226; 11.5%). This association was confirmed also when correcting for age (adjusted OR 2.07, 95% CI 1.06–4.08, *P* = 0.035) but not for BMI (*P* = 0.062).

As shown in Table 2, the adenomyosis was not associated with an increased risk of operative delivery, postpartum hemorrhage, need for manual removal of the placenta, and placental remnants after delivery.

## 4 DISCUSSION

To our knowledge, this is the first study reporting adenomyotic signs and their changes during pregnancy. The most commonly reported sign in the first trimester, i.e. the globular aspect of the uterus, which was reported in 79% of women with adenomyosis, progressively disappeared during the pregnancy, being present in 38% women in the second trimester and in only 2% in the third trimester. It was again visible in the postpartum period in 77% of women (30/39 with the globular sign in the first trimester without miscarriage and with postpartum evaluation available).

Moreover, this study showed an association between adenomyosis and adverse pregnancy outcomes, in particular with miscarriage in the first trimester, as previously reported in a review by Horton et al.^16^

Our study showed also an increased risk of CS in women with adenomyosis compared with controls, similar to what has been previously reported. This was confirmed also when correcting for age, which is a known risk factor for CS but not for BMI. Furthermore, in women with adenomyosis we found a trend towards preterm birth (*P* = 0.05), which was reported to be associated with adenomyosis in other studies.^10^, ^16^

The main strength of the present study is its originality: it is the first in the literature to evaluate how the signs of adenomyosis, usually seen in non‐pregnant women, can be identified in the first 12 weeks of gestation and how they change during pregnancy. This study confirmed the association between adenomyosis and adverse obstetric outcomes, although this association should be treated with caution.

The present study also presents several limitations. First, it is a monocentric retrospective study including only patients with known obstetric outcomes with a small sample size, in particular considering women with adenomyosis, which is a rare condition. In fact, we could not perform any further sub‐analysis according to the signs that were present at the first ultrasound. Although the rate of spontaneous and assisted reproductive technology pregnancies did not differ between the groups (see Table S1), we did not stratify the analysis according to the mode of conception because of the small sample size, possibly introducing a bias in pregnancy outcomes. Moreover, because of the retrospective nature of the study, some anamnestic information (i.e. history of preterm birth or recurrent miscarriages) was not available for analysis.

Despite this, we have shown that these signs are clinically relevant because adenomyosis resulted associated with an increased risk of miscarriage in the first trimester, as previously reported. However, well‐designed multicenter prospective studies with a larger sample size are needed to confirm our results.

Our study showed that, when assessed systematically, several typical signs of adenomyosis can be identified in a pregnant uterus within 12 weeks of conception. They progressively disappear during the pregnancy as a result of the progressive enlargement of the uterus and the complete anatomical adaptation of the uterus to the pregnancy with the myometrium becoming thinner and thinner with advancing gestation. The myometrial thickness, however, was still abnormal in the second trimester for almost half (20/52) of those having the globular sign at the first evaluation but then the sign progressively disappeared.

It is also possible that the high levels of progesterone in pregnancy might remodel the myometrial fibers and contribute to the progressive disappearance of this sign in the postpartum period.^17^ The levonorgestrel‐releasing intrauterine system can be successfully used to treat adenomyosis by causing a reduction of the myometrial junctional zone thickness and uterine volume.^18^, ^19^, ^20^ Other progestins are used to treat adenomyosis because they determine the decidualization and then atrophy of endometrial tissue, causing mild hypoestrogenism and having antiproliferative and anti‐inflammatory effects.^21^ It can be hypothesized that the high level of progesterone and the increase in the uterus size act simultaneously to determine a remodeling of the myometrial fibers throughout pregnancy.

Despite their progressive disappearance, the presence of signs of adenomyosis can be clinically relevant as it was associated with an increased risk of miscarriage in the first trimester as previously reported. The strength of this association in our cohort should be seen with caution because of the mean higher maternal age in women with adenomyosis, although this association was confirmed when considering age as a covariate.

No data on the relationship between miscarriage and adenomyosis have been published after normal conception according to the meta‐analysis performed by Horton et al.:^16^ in our cohort we confirmed this association also when considering only women with normal conception, as explained by a theory of suboptimal implantation and early development in women with adenomyosis. Therefore, our hypothesis is that adenomyosis per se might have an influence on implantation because of the molecular alterations present in the endometrium of women with adenomyosis,^21^, ^22^ including altered sex steroid hormone pathway, increased inflammatory markers and oxidative stress, reduced expression of implantation markers, and lack of expression of adhesion molecules, all resulting in impaired implantation.^23^

Despite being already described in younger women with reproductive disorders,^8^ we describe how signs of adenomyosis can be evaluated during pregnancy but, because of the small sample size and retrospective nature of the study, the presence of these signs does not imply any changes in clinical care at this time and should still be treated with caution. Further prospective studies are needed to confirm our findings and perform correlation analysis between changes of sonographic signs, and maternal and fetal outcomes.

In conclusion, sonographic signs of adenomyosis could be identified in pregnancy: they progressively disappear through gestation and become visible again in the postpartum period. These signs have a clinical relevance because adenomyosis is associated with an increased risk of miscarriage in the first trimester.

Further well‐designed prospective clinical studies with a multicentric larger sample size would be needed to confirm our results.

## AUTHOR CONTRIBUTIONS

ALM, EB, and CA conceived the original idea for this study. EB, FGS, and ALM designed the study. ALM and FGS developed the analysis plan. EB and MD extracted the data and prepared the data sets. FGS analyzed the data and conducted the literature searches. FGS, EB, and FF helped to identify previous work and gave the clinical interpretation. FGS, EB, and ALM wrote the first draft of the paper. All authors were involved in interpreting the findings and revising drafts and agreeing the final version.

## CONFLICT OF INTEREST

All authors have no conflicts of interest to disclose.

## Supporting information


Table S1

## Data Availability

Research data are not shared.
