# Supplementary material for: How adenomyosis changes throughout pregnancy: A retrospective cohort study
Source: Int J Gynaecol Obstet. 2022 Aug 17;160(3):856–63. doi: 10.1002/ijgo.14383 (PMC10087478; doi:10.1002/ijgo.14383)
Supplement: Supplementary file 1 — Table S1 [file IJGO-160-856-s001.docx]

**Supplementary Table 1**- General characteristics of the total population according to the presence or absence of adenomyosis.

|  | **Patients with adenomyosis (n=66)** | **Controls**  **(n=188)** | **p-value** |
| --- | --- | --- | --- |
| Maternal Age (yrs), *median (IQR)* | **38.0 (34-41)** | **35.0 (33-39)** | ***0.011*** |
| Maternal BMI (Kg/m^2^), *median (IQR)* | **22.6 (20.3-26.4)** | **21.1 (19.7-23.4)** | ***0.017*** |
| Pregnancy after IVF, *n(%)* | 10 (16,1%) | 26 (14.1%) | 0.689 |
| Nulliparous, *n(%)* | 42 (63.6%) | 123 (65.4%) | 0.793 |
| Previous CS, *n%* | 7 (10.6%) | 21 (11.2%) | 0.900 |
